# Supplementary material for: Arf1 coordinates fatty acid metabolism and mitochondrial homeostasis
Source: Nat Cell Biol. 2023 Jul 3;25(8):1157–72. doi: 10.1038/s41556-023-01180-2 (PMC10415182; doi:10.1038/s41556-023-01180-2)
Supplement: Supplementary file 1 — Supplementary Data 1 relative to FACS gating strategy and its legend. [file 41556_2023_1180_MOESM1_ESM.pdf]

nature cell biology

Article

https://doi.org/10.1038/s41556-023-01180-2

# Arf1 coordinates fatty acid metabolism and mitochondrial homeostasis

In the format provided by the authors and unedited

# Supplementary data 1

**A**

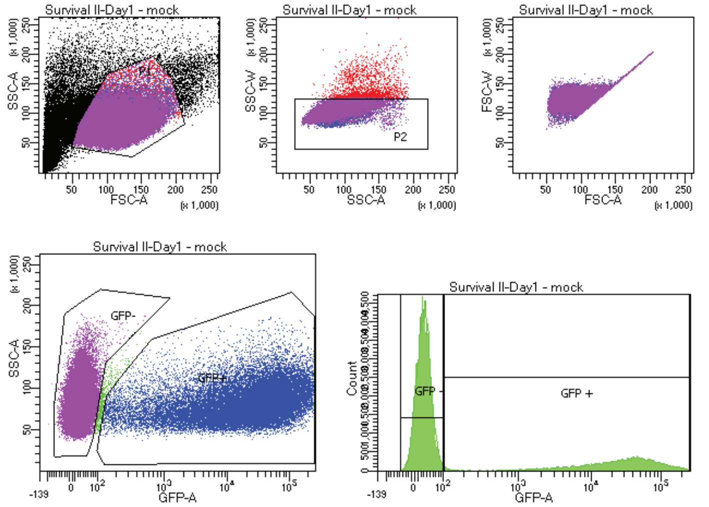


Tube: Day1 - mock

| Population | #Events | %Parent | %Total |
|---|---|---|---|
| All Events | 130,469 | #### | 100.0 |
| P1 | 97,536 | 74.8 | 74.8 |
| P2 | 95,478 | 97.9 | 73.2 |
| GFP+ | 30,496 | 31.9 | 23.4 |
| GFP- | 64,184 | 67.2 | 49.2 |
| GFP - | 63,859 | 66.9 | 48.9 |
| GFP + | 31,131 | 32.6 | 23.9 |

Experiment Name: GFP experiments
Specimen Name: Survival II
Tube Name: Day1 - mock
Record Date: Jun 2, 2021 11:35:39 AM
$OP: Mirjam
GUID: 474d7ecf-aa75-4e65-a4c4-c9613e8eea...

| Population | #Events | %Parent | GFP-A Min | GFP-A Max | GFP-A Median |
|---|---|---|---|---|---|
| GFP+ | 30,496 | 31.9 | 107 | 250,959 | 23,440 |
| GFP- | 64,184 | 67.2 | -72 | 515 | 22 |
| GFP - | 63,859 | 66.9 | -50 | 96 | 22 |
| GFP + | 31,131 | 32.6 | 101 | 251,695 | 22,523 |

**B**

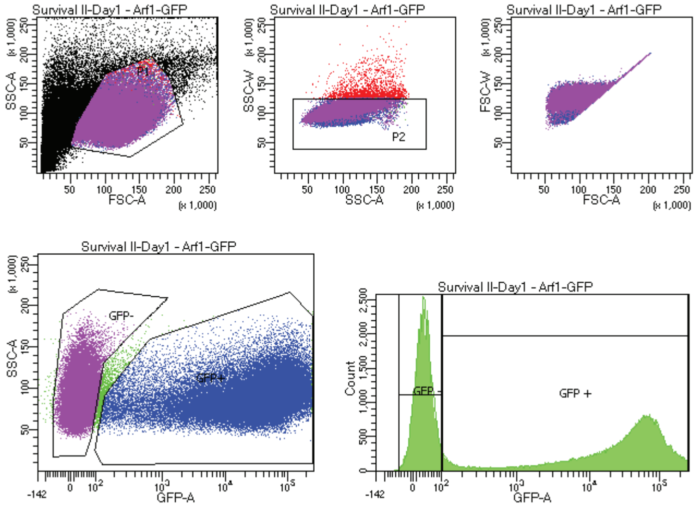


Tube: Day1 - Arf1-GFP

| Population | #Events | %Parent | %Total |
|---|---|---|---|
| All Events | 149,970 | #### | 100.0 |
| P1 | 94,429 | 63.0 | 63.0 |
| P2 | 92,418 | 97.9 | 61.6 |
| GFP+ | 49,802 | 53.9 | 33.2 |
| GFP- | 40,711 | 44.1 | 27.1 |
| GFP - | 39,716 | 43.0 | 26.5 |
| GFP + | 51,506 | 55.7 | 34.3 |

Experiment Name: GFP experiments
Specimen Name: Survival II
Tube Name: Day1 - Arf1-GFP
Record Date: Jun 2, 2021 11:40:44 AM
$OP: Mirjam
GUID: ba6ab357-0fcd-410c-8a80-88f758eb1b...

| Population | #Events | %Parent | GFP-A Min | GFP-A Max | GFP-A Median |
|---|---|---|---|---|---|
| GFP+ | 49,802 | 53.9 | 106 | 250,951 | 37,712 |
| GFP- | 40,711 | 44.1 | -72 | 414 | 28 |
| GFP - | 39,716 | 43.0 | -50 | 96 | 27 |
| GFP + | 51,506 | 55.7 | 101 | 251,708 | 35,658 |

**C**

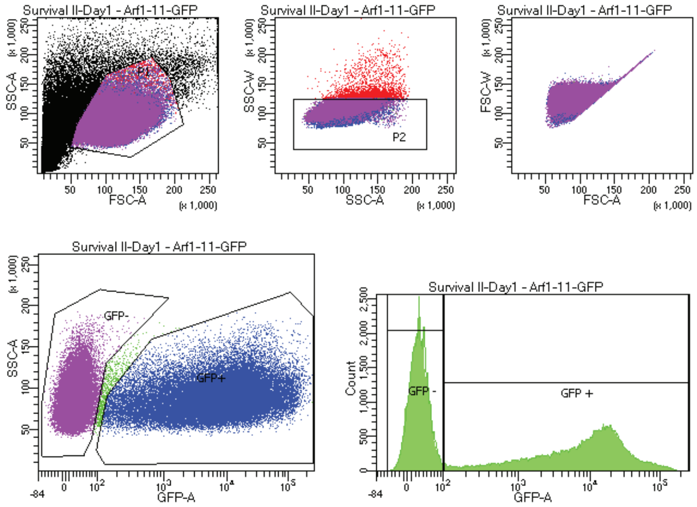


Tube: Day1 - Arf1-11-GFP

| Population | #Events | %Parent | %Total |
|---|---|---|---|
| All Events | 150,294 | #### | 100.0 |
| P1 | 88,509 | 58.9 | 58.9 |
| P2 | 85,740 | 96.9 | 57.0 |
| GFP+ | 45,007 | 52.5 | 29.9 |
| GFP- | 40,057 | 46.7 | 26.7 |
| GFP - | 39,751 | 46.4 | 26.4 |
| GFP + | 45,873 | 53.5 | 30.5 |

Experiment Name: GFP experiments
Specimen Name: Survival II
Tube Name: Day1 - Arf1-11-GFP
Record Date: Jun 2, 2021 11:48:55 AM
$OP: Mirjam
GUID: e11d7632-4fac-4753-b8e2-d2120121d...

| Population | #Events | %Parent | GFP-A Min | GFP-A Max | GFP-A Median |
|---|---|---|---|---|---|
| GFP+ | 45,007 | 52.5 | 112 | 245,797 | 9,346 |
| GFP- | 40,057 | 46.7 | -66 | 495 | 23 |
| GFP - | 39,751 | 46.4 | -50 | 96 | 23 |
| GFP + | 45,873 | 53.5 | 101 | 245,797 | 9,036 |

# Supplementary information

## Supplementary legends

**Supplementary data 1. Gating strategy for FACS sorting and GFP measurement in HeLa *ARF1 KO* (related to ED figure 3).**

Gating strategy to measure cell survival of GFP-positive cells in ARF1 KO HeLa alpha cells transfected with empty vector - mock (A), Arf1-GFP (B) or Arf1-11-GFP (C) plasmids. To exclude debris from the initial cell population, forward (FSC) versus side scatter (SSC) gating was applied (population P1). Single cells were determined by using SSC area vs. SSC width (SSC-A/SSC-W) gating and by FSC-A/FSC-W gating (population P2). From the single cell population, GFP+ cells were determined by gating GFP-A/SSC-A density plots (population GFP+). 100,000 single cells were measured and the percentage of GFP+ cells were determined in each sample (%Parent).
