# Supplementary material for: Arf1 coordinates fatty acid metabolism and mitochondrial homeostasis
Source: Nat Cell Biol. 2023 Jul 3;25(8):1157–72. doi: 10.1038/s41556-023-01180-2 (PMC10415182; doi:10.1038/s41556-023-01180-2)

WB related to Figure 3A

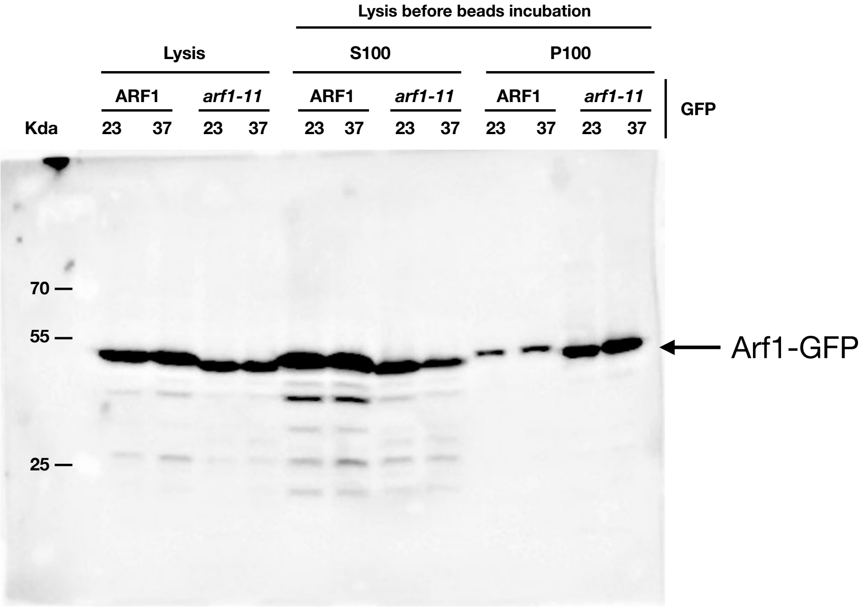

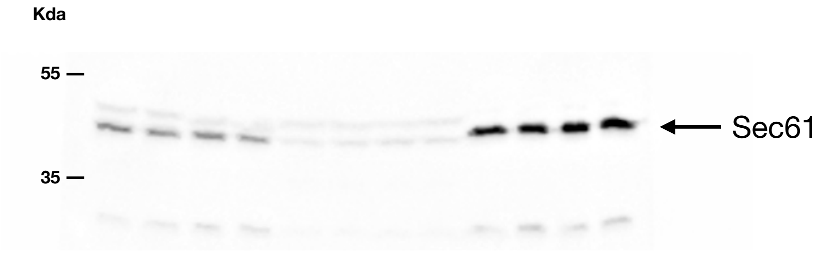

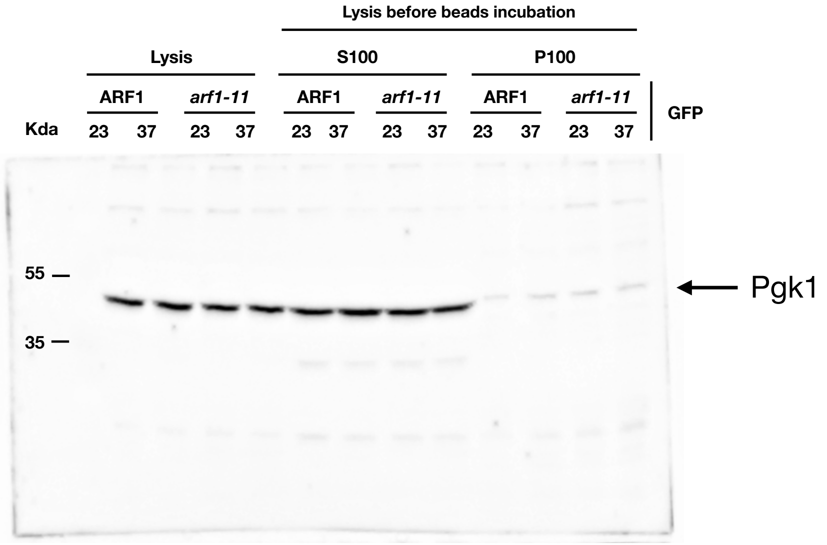

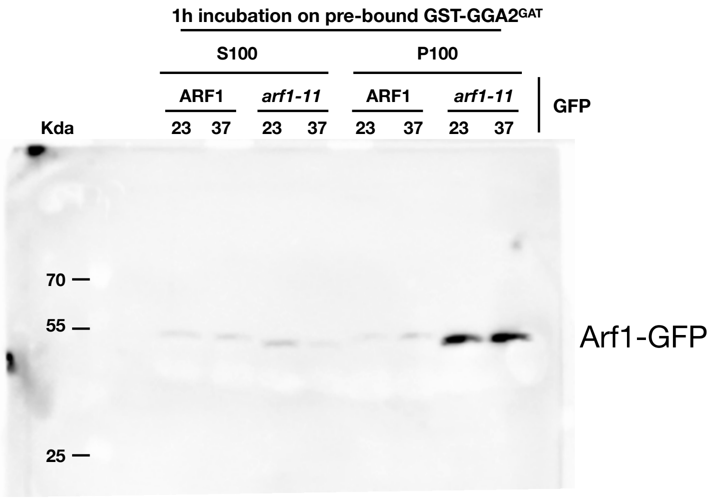

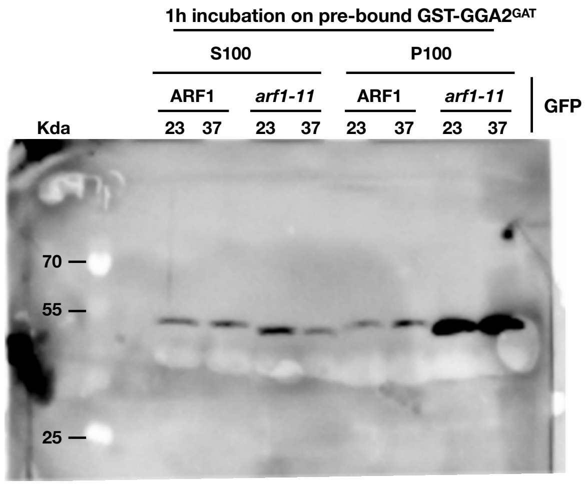

WB related to Figure 3B

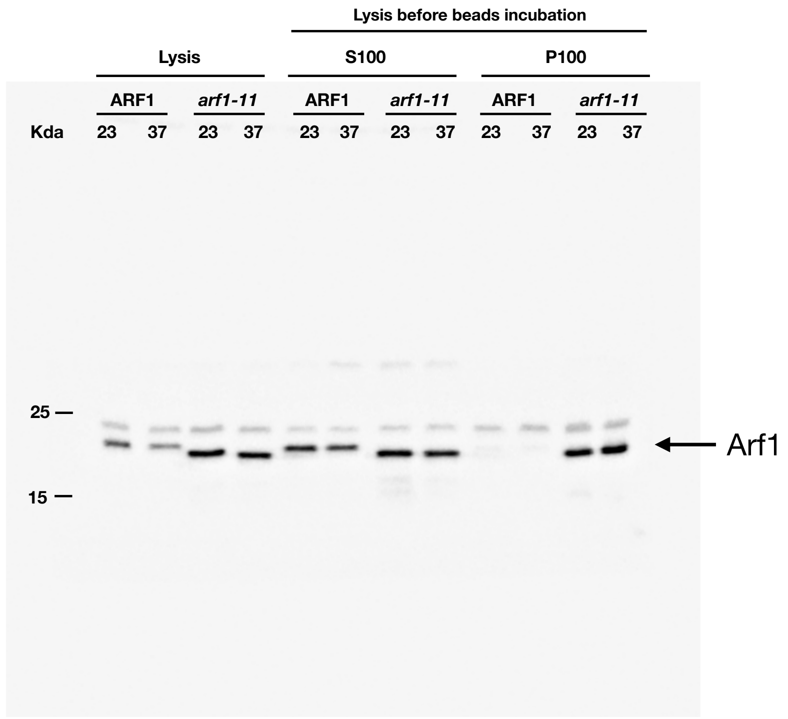

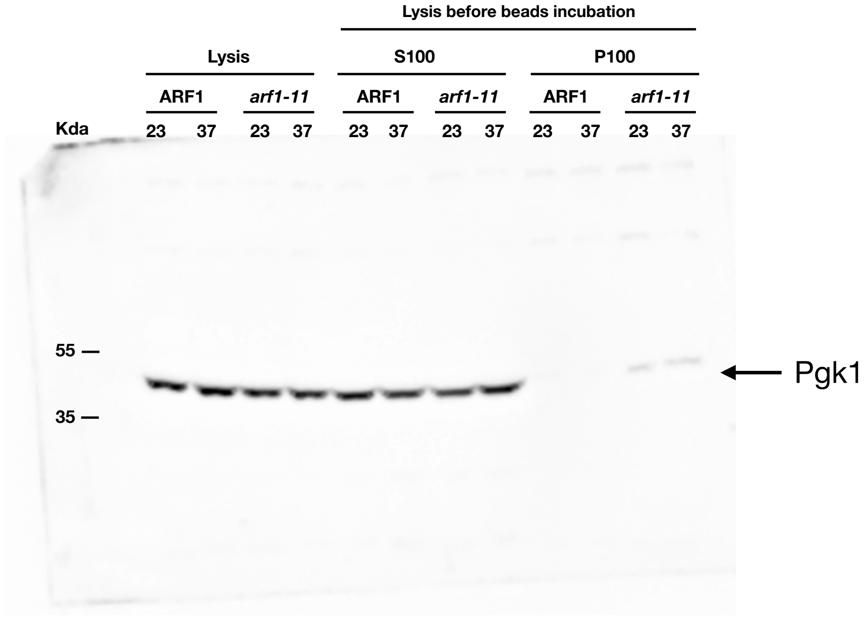

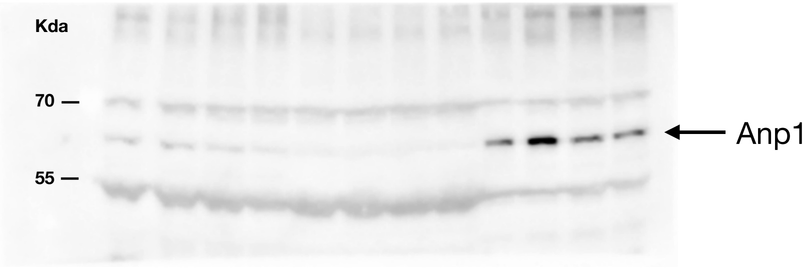

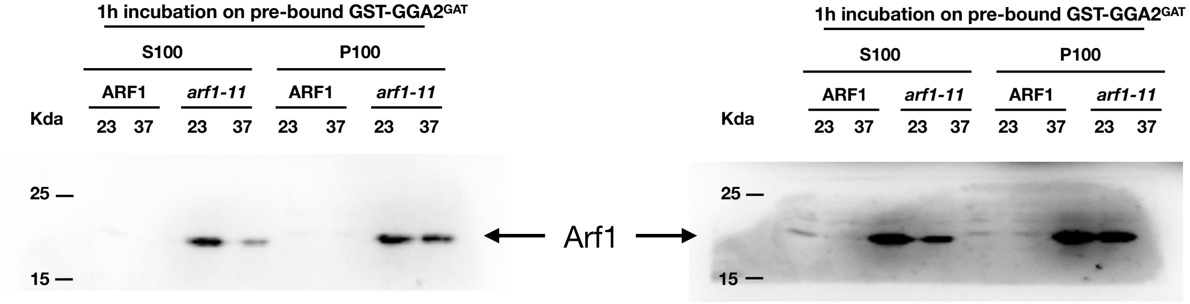

WB related to Figure 7B

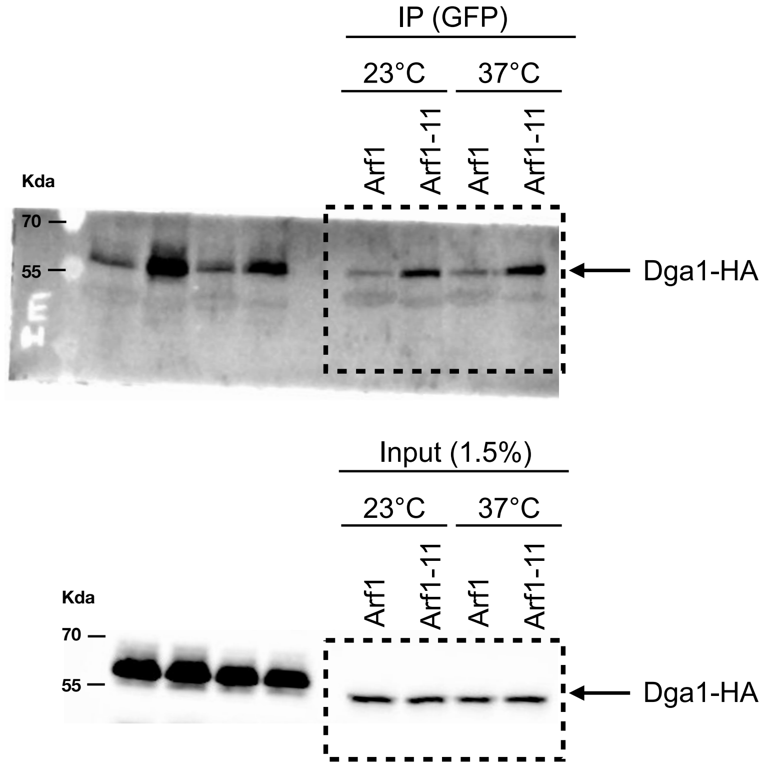

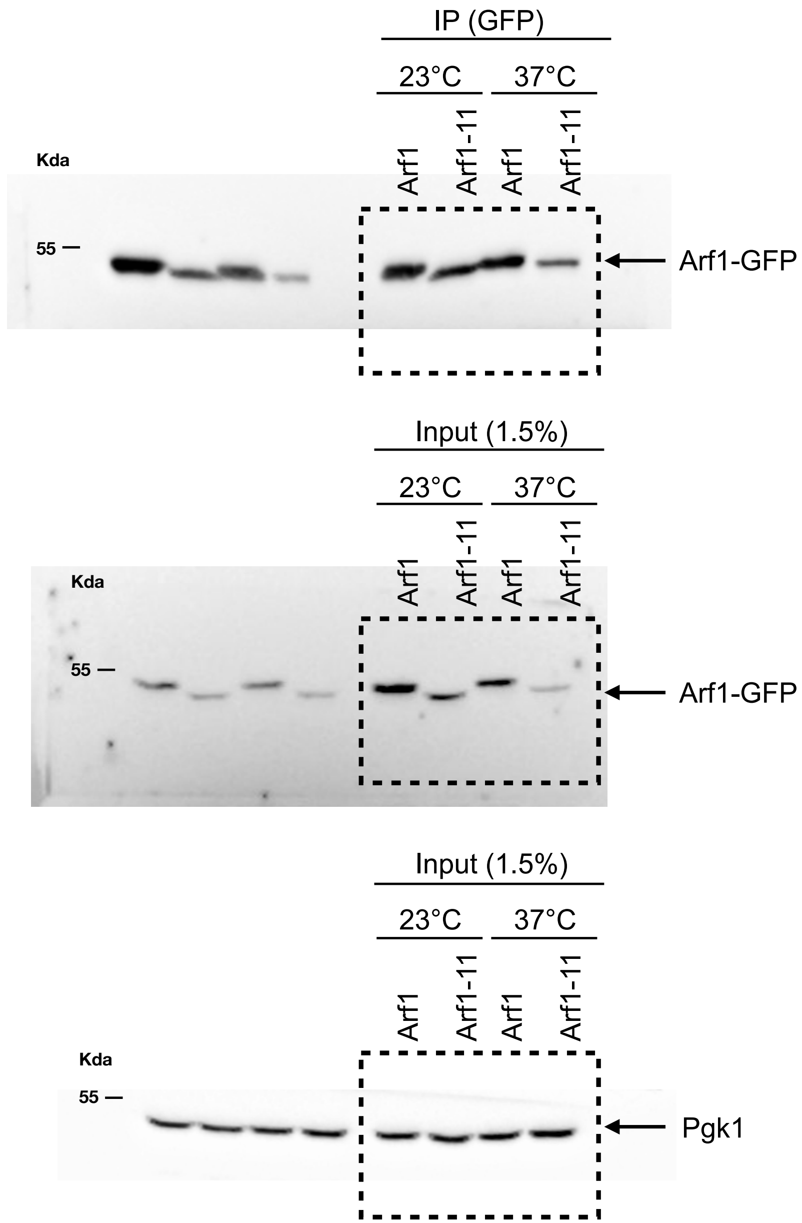

WB related to Figure 7D

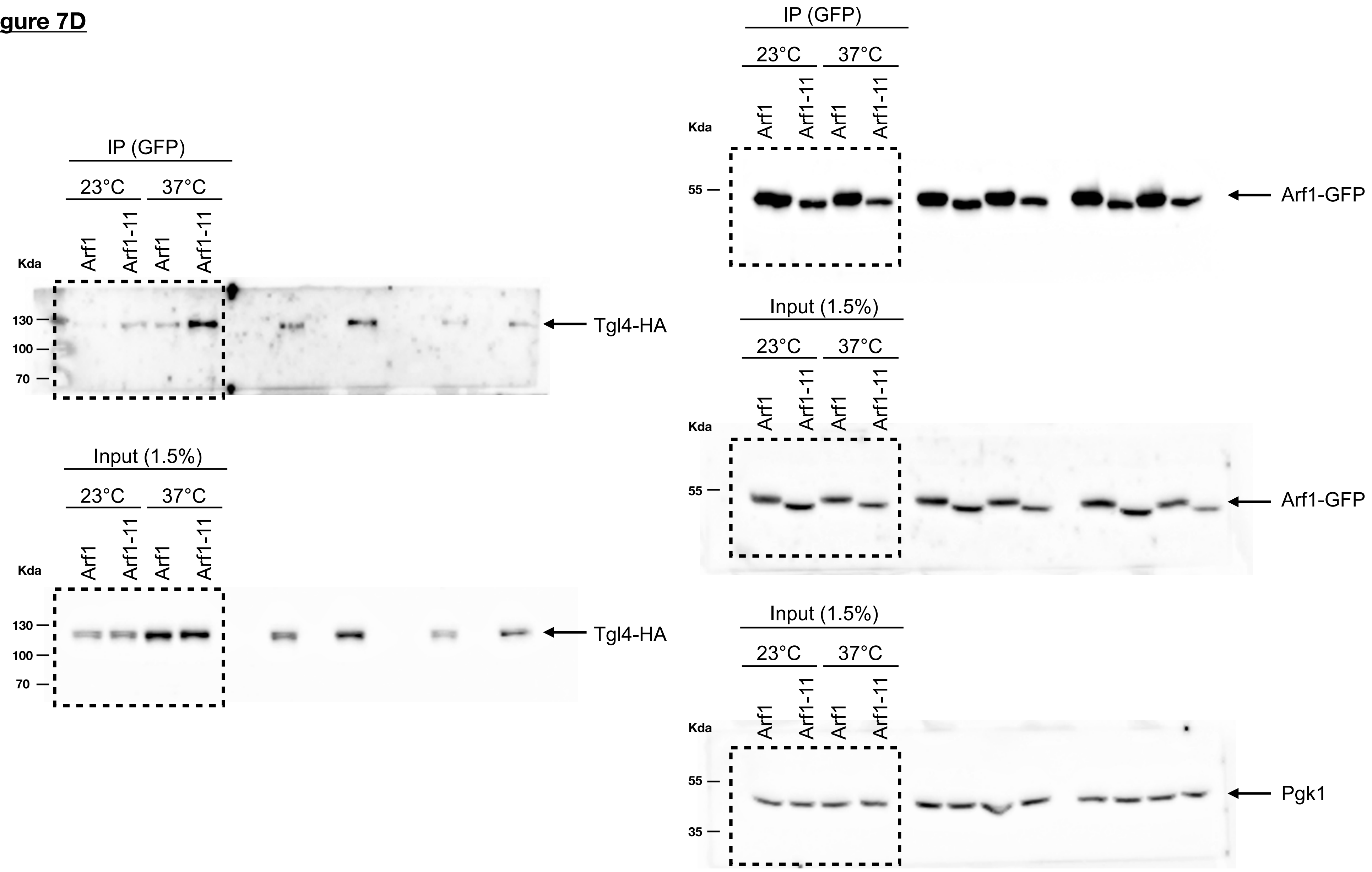

WB related to Figure 7E

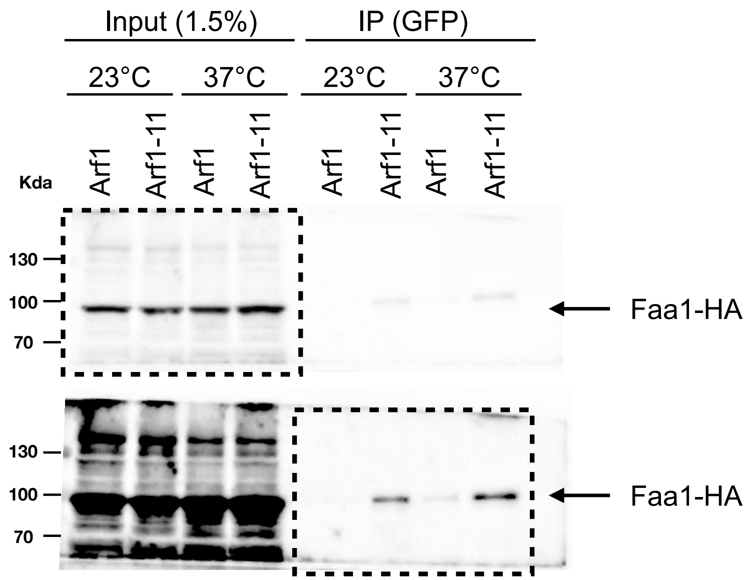

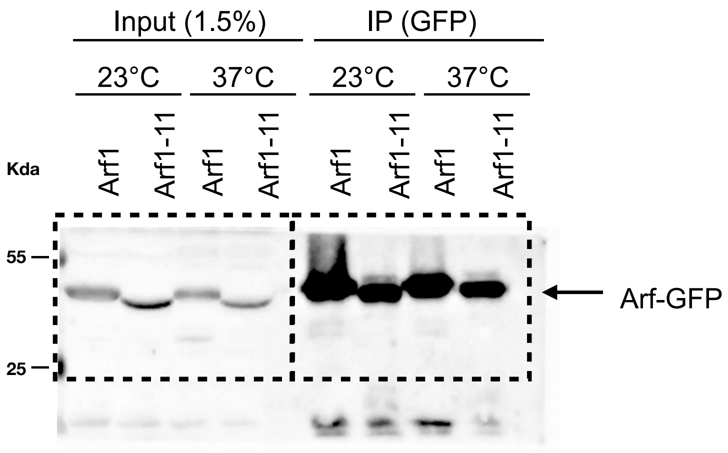

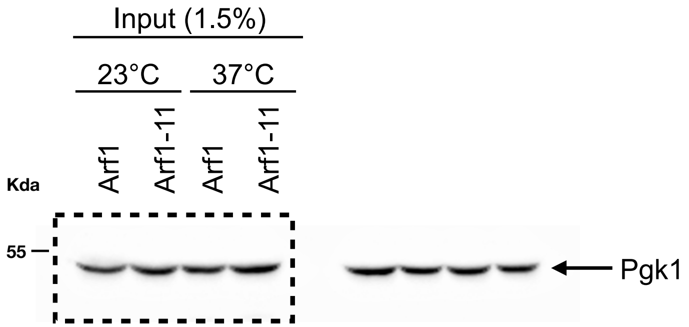

WB related to Figure 7H

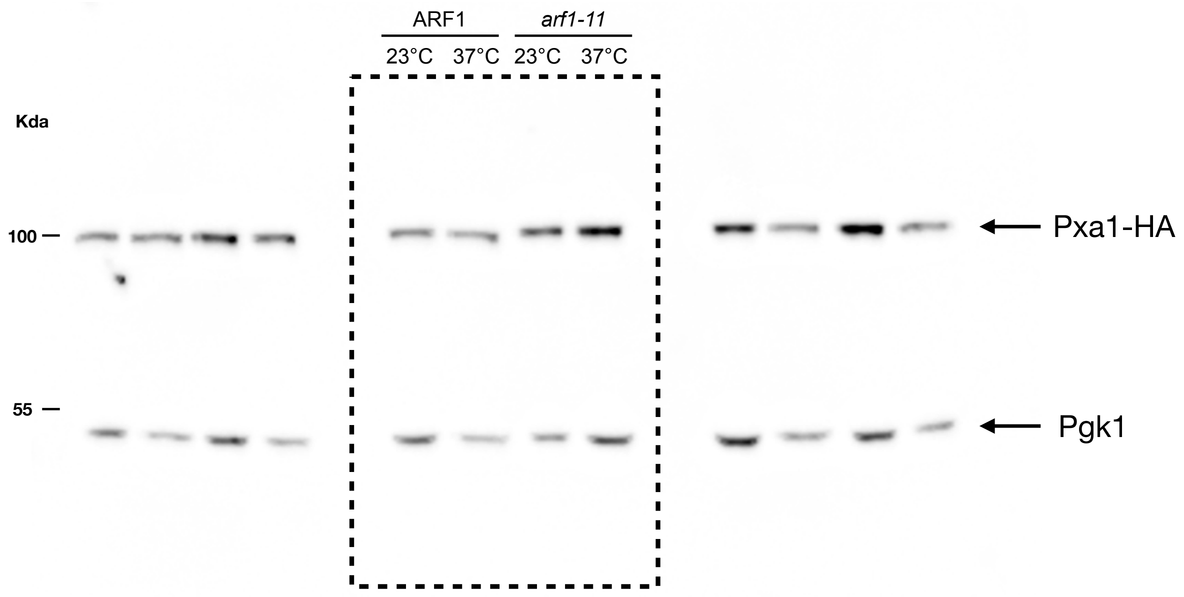

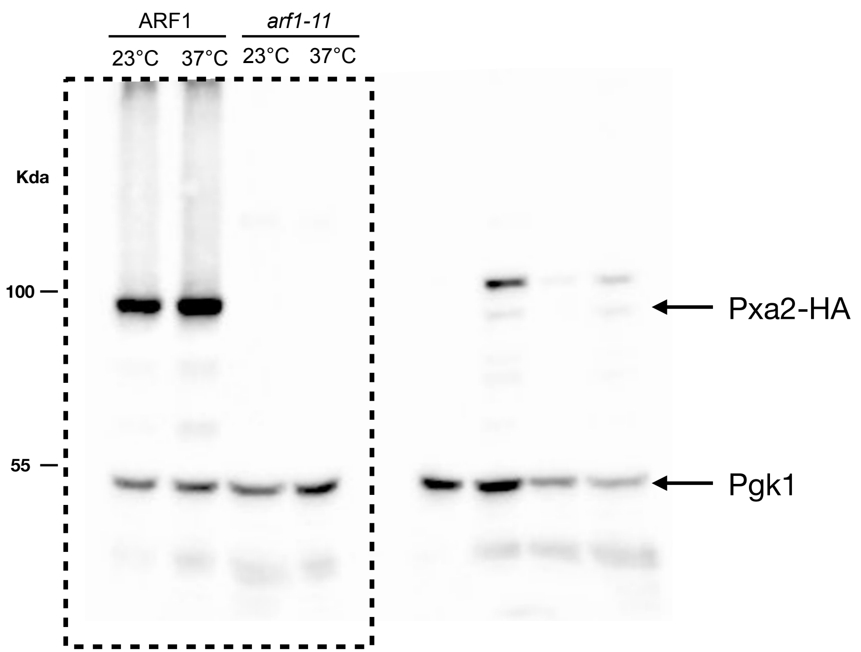

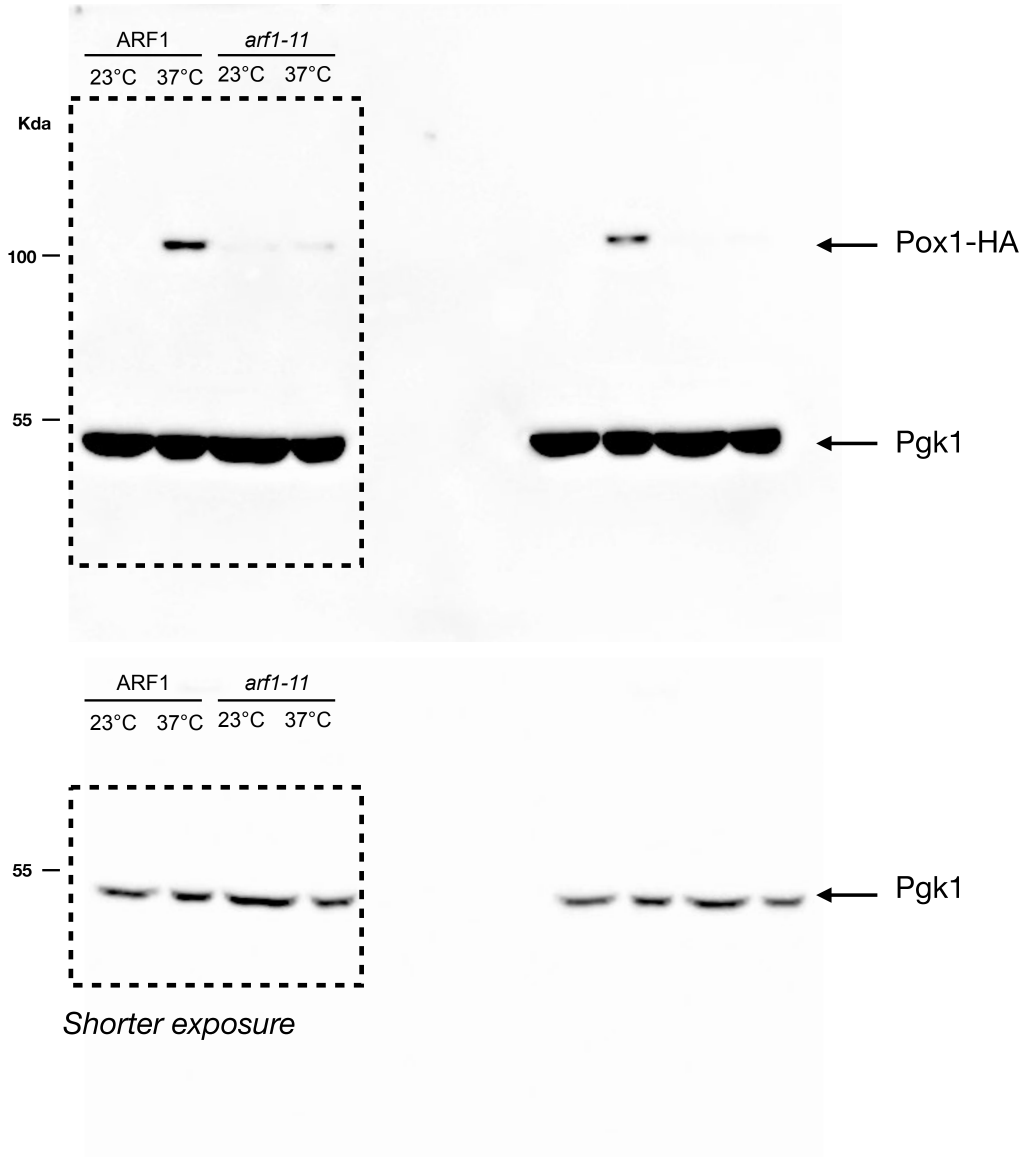
ARF1
arf1-11
23°C
37°C
23°C
37°C
Kda
100
55
Pox1-HA
Pgk1
ARF1
arf1-11
23°C
37°C
23°C
37°C
55
Pgk1
Shorter exposure

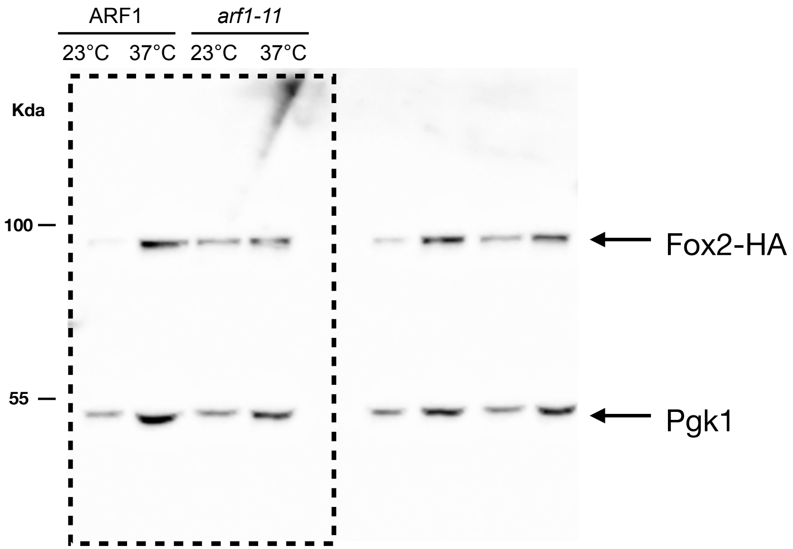
ARF1
arf1-11
23°C
37°C
23°C
37°C
Kda
100
55
Fox2-HA
Pgk1

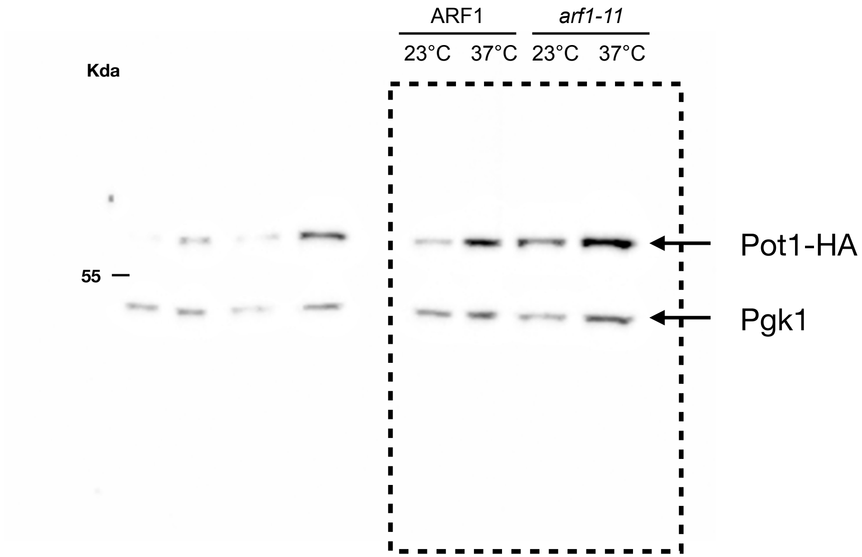
ARF1
arf1-11
23°C 37°C 23°C 37°C
Kda
55
Pot1-HA
Pgk1

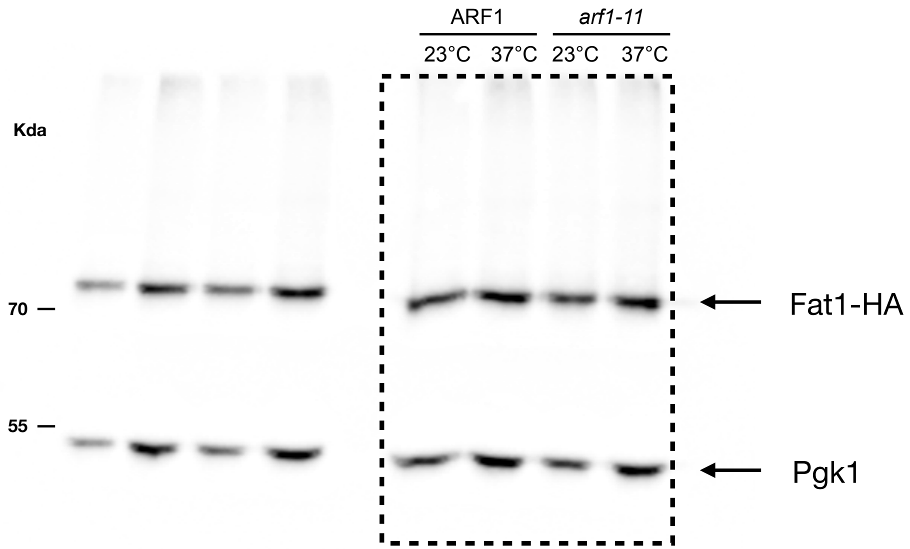
ARF1
arf1-11
23°C 37°C 23°C 37°C
Kda
70
55
Fat1-HA
Pgk1

WB related to ED figure 3B

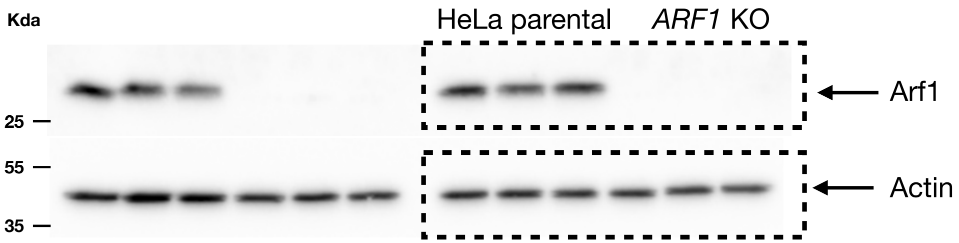

WB related to ED figure 3C

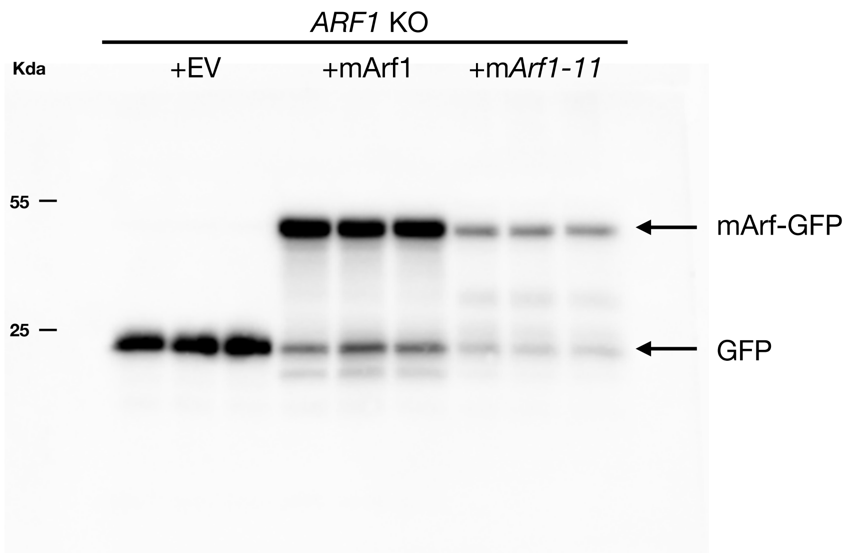

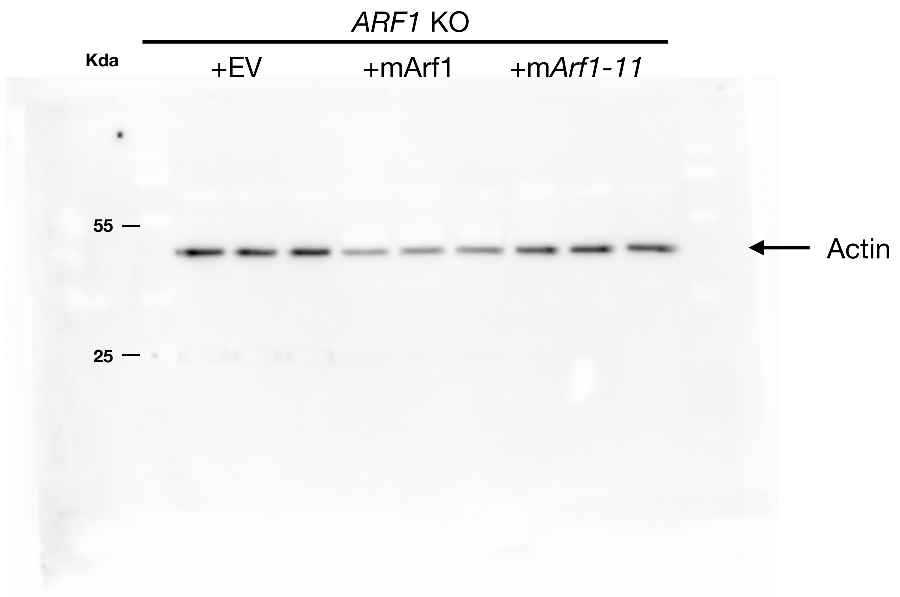

WB related to ED figure 5F

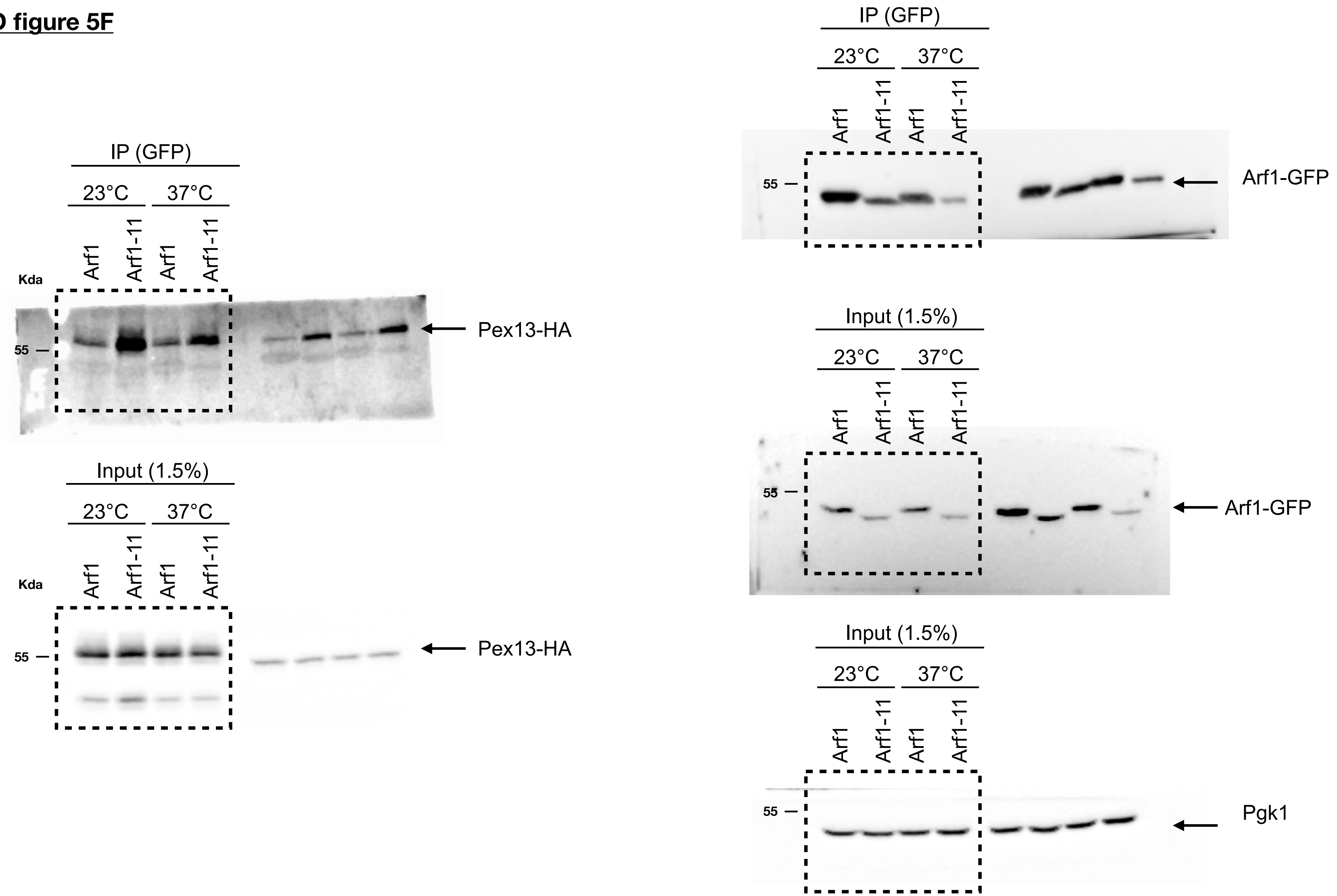

Supplement: Source Data Figs. 3 and 7, and Extended Data Figs. 3 and 5 — Unprocessed western blots for all relevant figures. [file 41556_2023_1180_MOESM21_ESM.pdf]
